# (*S*)-2-Benzyl-*N*-(2,6-diisopropyl­phen­yl)-1,2,3,4-tetra­hydro­isoquinoline-3-carboxamide

**DOI:** 10.1107/S1600536811012554

**Published:** 2011-04-13

**Authors:** Tricia Naicker, Thavendran Govender, Hendrik G. Kruger, Glenn E. M. Maguire

**Affiliations:** a School of Pharmacy and Pharmacology, University of KwaZulu-Natal, Durban 4000, South Africa; b School of Chemistry, University of KwaZulu-Natal, Durban 4000, South Africa

## Abstract

The asymmetric unit of the title compound, C_29_H_34_N_2_O, contains two mol­ecules in which the N-containing six-membered rings assume different conformations *viz*. half-chair and envelope. Inter­molecular N—H⋯O hydrogen bonding *via* the amide groups cross-link the mol­ecules in the crystal structure.

## Related literature

The title compound is a precursor to novel *N*-oxide type organocatalysts, see: Naicker *et al.* (2010 ▶). For a related structure, see: Naicker *et al.* (2011 ▶).
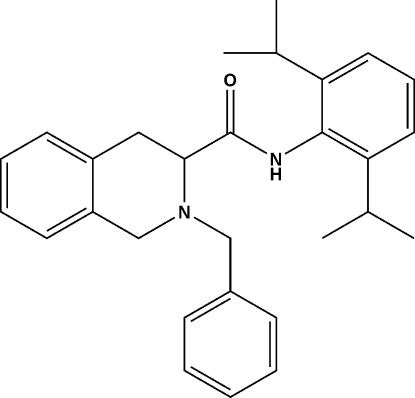


## Experimental

### 

#### Crystal data

C_29_H_34_N_2_O
*M* *_r_* = 426.58
Monoclinic, 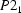

*a* = 9.493 (3) Å
*b* = 12.459 (5) Å
*c* = 21.280 (8) Å
β = 102.241 (7)°
*V* = 2459.8 (16) Å^3^
*Z* = 4
Mo *K*α radiation
μ = 0.07 mm^−1^
*T* = 173 K
0.35 × 0.06 × 0.05 mm

#### Data collection

Bruker Kappa DUO APEXII diffractometer
Absorption correction: multi-scan (*SADABS*; Bruker, 2006 ▶) *T*_min_ = 0.976, *T*_max_ = 0.997
16565 measured reflections
4932 independent reflections
2620 reflections with *I* > 2σ(*I*)
*R*_int_ = 0.093

#### Refinement

*R*[*F* ^2^ > 2σ(*F* ^2^)] = 0.057
*wR*(*F* ^2^) = 0.146
*S* = 0.98
4932 reflections
577 parameters
1 restraint
H-atom parameters constrained
Δρ_max_ = 0.24 e Å^−3^
Δρ_min_ = −0.18 e Å^−3^

### 

Data collection: *APEX2* (Bruker, 2006 ▶); cell refinement: *SAINT* (Bruker, 2006 ▶); data reduction: *SAINT*; program(s) used to solve structure: *SHELXS97* (Sheldrick, 2008 ▶); program(s) used to refine structure: *SHELXL97* (Sheldrick, 2008 ▶); molecular graphics: *OLEX2* (Dolomanov *et al.*, 2009 ▶); software used to prepare material for publication: *SHELXL97*.

## Supplementary Material

Crystal structure: contains datablocks I, global. DOI: 10.1107/S1600536811012554/hg5021sup1.cif

Structure factors: contains datablocks I. DOI: 10.1107/S1600536811012554/hg5021Isup2.hkl

Additional supplementary materials:  crystallographic information; 3D view; checkCIF report

## Figures and Tables

**Table 1:** Hydrogen-bond geometry (Å, °)

*D*—H⋯*A*	*D*—H	H⋯*A*	*D*⋯*A*	*D*—H⋯*A*
N2*A*—H2*A*⋯O1*B*^i^	0.88	2.15	2.900 (6)	142

Symmetry code: (i) 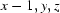.

**Fig. 1.**
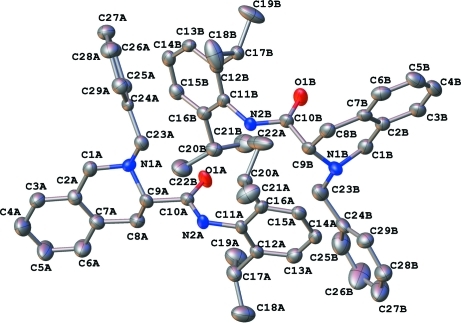
The molecular structure of the title compound with the atom numbering scheme. Displacement ellipsoids are drawn at the 40% probability level. Hydrogen atoms have been omitted for clarity.

**Fig. 2.**
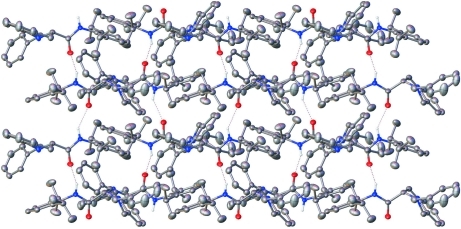
A partial projection of the title compound, viewed along [001] plane.

## supplementary crystallographic information

### Comment

The title compound is a precursor in the synthesis of novel chiral catalysts containing a tetrahydroisoquinoline framework (TIQ). Upon oxidation of the secondary amine, the *N*-oxide form of this compound and its derivatives are currently being tested as novel organocatalysts for asymmetric allylation reactions (Naicker *et al.* 2010).

The structure has two molecules in the asymmetric unit (Fig. 1). These molecules are linked *via* various intermolecular short contact interactions (2.01–2.83 Å). The crystal packing reveals that a hydrogen bond *via* the amide groups N2A—H2A···O1B link the molecules together resulting in a one-dimensional sheet along the *c* axis (Fig. 2), also see (Naicker *et al.* 2011)

From the crystal structure it is evident that the *N*-containing six membered rings assume different conformations for the two molecules in the asymmetric unit (Fig. 1). The ring containing N1A adopts a half chair conformation while N1B exists as a half boat conformation.

### Experimental

(*S*)-2-Benzyl-1,2,3,4-tetrahydroisoquinoline-3-carboxylic acid (1.5 g, 3.4 mmol) was dissolved in dry dichloromethane (15 ml) followed by the addition of triethylamine (2.0 eq.) and ethylchloroformate (1.2 eq.) which was stirred for 1 h at 0 degrees followed by the addition of 2,6-diisopropylaniline (1.1 eq.). The reaction mixture was then stirred at room temperature until no more starting material could be detected by TLC analysis (approximately 3 h). The reaction mixture was poured into water (30 equivalent volumes); the mixture was then extracted twice with dichloromethane (20 ml). The extracts were combined, dried over anhydrous magnesium sulfate and then concentrated to dryness affording the crude product which was purified by silica column chromatography (hexane:ethylacetate 80:20 *R*_f_ =0.6).

Melting point 418–420 K. [*α*]^20^_D_ +4.762 (*c* 0.14 in CHCl_3_).

IR (neat): 3286, 2961, 1676,1488, 746 cm^-1^.

^1^H NMR (400 MHz, CDCl_3_) δ 8.87 (s, 1H), 7.45 – 7.32 (m, 5H), 7.27 – 7.16 (m, 5H), 7.09 (t, *J* = 5.8 Hz, 3H), 3.92 (dt, *J* = 16.1, 13.2 Hz, 3H), 3.80 (dd, *J* = 7.2, 4.2 Hz, 1H), 3.68 (d, *J* = 13.7 Hz, 1H), 3.33 (dd, *J* = 15.4, 4.2 Hz, 1H), 3.18 (dd, *J* = 15.4, 7.2 Hz, 1H), 1.59 (s, 4H), 1.00 (dd, *J* = 20.3, 6.2 Hz, 13H).

^13^C NMR (101 MHz, CDCl_3_) δ 172.91, 145.83, 137.71, 135.85, 134.98, 131.15, 128.96, 128.76, 127.93, 127.90, 127.71, 127.43, 126.55, 126.17, 123.28, 77.34, 77.02, 76.70, 62.42, 60.85, 51.60, 29.76, 28.50, 23.72, 23.64.

Recrystallization from dichloromethane at room temperature afforded colourless crystals suitable for X-ray analysis.

The absolute configuration was confirmed by NMR studies.

### Refinement

All non-hydrogen atoms were refined anisotropically. All hydrogen atoms were positioned geometrically with C—H distances ranging from 0.95 Å to 1.00 Å and refined as riding on their parent atoms with *U*_iso_ (H) = 1.2 - 1.5 *U*_eq_ (C). With unmerged reflections the Flack *x* parameter equals to -1.3800 with e.s.d. 2.4120.

### Crystal data

**Table:**

C_29_H_34_N_2_O	*F*(000) = 920
*M**_r_* = 426.58	*D*_x_ = 1.152 Mg m^−^^3^
Monoclinic, *P*2_1_	Melting point: 419 K
Hall symbol: P 2yb	Mo *K*α radiation, λ = 0.71073 Å
*a* = 9.493 (3) Å	Cell parameters from 16565 reflections
*b* = 12.459 (5) Å	θ = 1.9–25.9°
*c* = 21.280 (8) Å	µ = 0.07 mm^−^^1^
β = 102.241 (7)°	*T* = 173 K
*V* = 2459.8 (16) Å^3^	Needle, colourless
*Z* = 4	0.35 × 0.06 × 0.05 mm

### Data collection

**Table:**

Bruker Kappa DUO APEXII diffractometer	4932 independent reflections
Radiation source: fine-focus sealed tube	2620 reflections with *I* > 2σ(*I*)
graphite	*R*_int_ = 0.093
0.5° φ scans and ω scans	θ_max_ = 25.9°, θ_min_ = 1.9°
Absorption correction: multi-scan (*SADABS*; Bruker, 2006)	*h* = −5→11
*T*_min_ = 0.976, *T*_max_ = 0.997	*k* = −15→15
16565 measured reflections	*l* = −25→25

### Refinement

**Table:**

Refinement on *F*^2^	Primary atom site location: structure-invariant direct methods
Least-squares matrix: full	Secondary atom site location: difference Fourier map
*R*[*F*^2^ > 2σ(*F*^2^)] = 0.057	Hydrogen site location: inferred from neighbouring sites
*wR*(*F*^2^) = 0.146	H-atom parameters constrained
*S* = 0.98	*w* = 1/[σ^2^(*F*_o_^2^) + (0.0643*P*)^2^] where *P* = (*F*_o_^2^ + 2*F*_c_^2^)/3
4932 reflections	(Δ/σ)_max_ < 0.001
577 parameters	Δρ_max_ = 0.24 e Å^−^^3^
1 restraint	Δρ_min_ = −0.18 e Å^−^^3^

### Special details

**Table:**

Experimental. Half sphere of data collected using the Bruker *SAINT* software package. Crystal to detector distance = 40 mm; combination of φ and ω scans of 0.5°, 50 s per °, 2 iterations.
Geometry. All e.s.d.'s (except the e.s.d. in the dihedral angle between two l.s. planes) are estimated using the full covariance matrix. The cell e.s.d.'s are taken into account individually in the estimation of e.s.d.'s in distances, angles and torsion angles; correlations between e.s.d.'s in cell parameters are only used when they are defined by crystal symmetry. An approximate (isotropic) treatment of cell e.s.d.'s is used for estimating e.s.d.'s involving l.s. planes.
Refinement. Refinement of *F*^2^ against ALL reflections. The weighted *R*-factor *wR* and goodness of fit *S* are based on *F*^2^, conventional *R*-factors *R* are based on *F*, with *F* set to zero for negative *F*^2^. The threshold expression of *F*^2^ > σ(*F*^2^) is used only for calculating *R*-factors(gt) *etc*. and is not relevant to the choice of reflections for refinement. *R*-factors based on *F*^2^ are statistically about twice as large as those based on *F*, and *R*- factors based on ALL data will be even larger.

### Fractional atomic coordinates and isotropic or equivalent isotropic displacement parameters (Å^2^)

**Table:**

	*x*	*y*	*z*	*U*_iso_*/*U*_eq_	
O1A	0.4520 (4)	0.3788 (3)	0.7605 (2)	0.0553 (11)	
N1A	0.3190 (4)	0.1660 (3)	0.7484 (2)	0.0400 (11)	
N2A	0.2265 (4)	0.4455 (3)	0.7444 (2)	0.0397 (11)	
H2A	0.1342	0.4309	0.7402	0.048*	
C1A	0.2497 (6)	0.0682 (4)	0.7640 (3)	0.0458 (14)	
H1A1	0.3039	0.0059	0.7526	0.055*	
H1A2	0.1512	0.0650	0.7369	0.055*	
C2A	0.2389 (6)	0.0573 (4)	0.8331 (3)	0.0428 (13)	
C3A	0.1910 (6)	−0.0390 (4)	0.8550 (3)	0.0527 (16)	
H3A	0.1676	−0.0975	0.8259	0.063*	
C4A	0.1770 (8)	−0.0512 (6)	0.9181 (4)	0.077 (2)	
H4A	0.1432	−0.1169	0.9322	0.092*	
C5A	0.2131 (9)	0.0344 (5)	0.9607 (3)	0.082 (2)	
H5A	0.2061	0.0268	1.0043	0.098*	
C6A	0.2595 (7)	0.1308 (5)	0.9393 (3)	0.0652 (18)	
H6A	0.2835	0.1893	0.9682	0.078*	
C7A	0.2710 (6)	0.1420 (5)	0.8754 (3)	0.0495 (15)	
C8A	0.3150 (6)	0.2485 (4)	0.8519 (3)	0.0505 (15)	
H8A1	0.4212	0.2555	0.8639	0.061*	
H8A2	0.2725	0.3072	0.8732	0.061*	
C9A	0.2653 (5)	0.2596 (4)	0.7787 (3)	0.0422 (14)	
H9A	0.1575	0.2603	0.7671	0.051*	
C10A	0.3221 (6)	0.3654 (4)	0.7591 (3)	0.0425 (14)	
C11A	0.2686 (5)	0.5540 (4)	0.7353 (3)	0.0375 (13)	
C12A	0.3111 (5)	0.6185 (4)	0.7897 (3)	0.0412 (13)	
C13A	0.3565 (6)	0.7229 (4)	0.7806 (3)	0.0494 (15)	
H13A	0.3881	0.7682	0.8168	0.059*	
C14A	0.3559 (5)	0.7612 (4)	0.7194 (3)	0.0457 (14)	
H14A	0.3865	0.8325	0.7139	0.055*	
C15A	0.3109 (5)	0.6961 (4)	0.6664 (3)	0.0433 (14)	
H15A	0.3085	0.7239	0.6246	0.052*	
C16A	0.2686 (5)	0.5892 (4)	0.6732 (3)	0.0392 (13)	
C17A	0.3121 (6)	0.5779 (5)	0.8585 (3)	0.0535 (16)	
H17A	0.2544	0.5101	0.8537	0.064*	
C18A	0.2384 (8)	0.6556 (7)	0.8972 (3)	0.085 (2)	
H18A	0.1420	0.6734	0.8724	0.128*	
H18B	0.2959	0.7214	0.9061	0.128*	
H18C	0.2304	0.6218	0.9379	0.128*	
C19A	0.4627 (7)	0.5488 (6)	0.8949 (3)	0.082 (2)	
H19A	0.5068	0.4990	0.8690	0.122*	
H19B	0.4570	0.5146	0.9357	0.122*	
H19C	0.5215	0.6139	0.9033	0.122*	
C20A	0.2224 (5)	0.5200 (4)	0.6144 (3)	0.0446 (14)	
H20A	0.2029	0.4466	0.6296	0.054*	
C21A	0.0830 (7)	0.5606 (6)	0.5725 (3)	0.078 (2)	
H21A	0.0555	0.5142	0.5347	0.116*	
H21B	0.0968	0.6341	0.5585	0.116*	
H21C	0.0066	0.5597	0.5970	0.116*	
C22A	0.3397 (7)	0.5089 (7)	0.5768 (4)	0.100 (3)	
H22A	0.3054	0.4635	0.5390	0.150*	
H22B	0.4250	0.4761	0.6040	0.150*	
H22C	0.3648	0.5800	0.5628	0.150*	
C23A	0.2944 (6)	0.1801 (4)	0.6783 (3)	0.0432 (13)	
H23A	0.3346	0.2501	0.6688	0.052*	
H23B	0.1894	0.1813	0.6601	0.052*	
C24A	0.3627 (6)	0.0920 (4)	0.6462 (3)	0.0405 (14)	
C25A	0.4969 (5)	0.0471 (4)	0.6742 (3)	0.0423 (13)	
H25A	0.5460	0.0692	0.7158	0.051*	
C26A	0.5571 (6)	−0.0294 (4)	0.6408 (3)	0.0485 (15)	
H26A	0.6468	−0.0607	0.6605	0.058*	
C27A	0.4912 (7)	−0.0610 (5)	0.5806 (3)	0.0571 (16)	
H27A	0.5355	−0.1132	0.5585	0.069*	
C28A	0.3621 (7)	−0.0179 (5)	0.5520 (3)	0.0591 (17)	
H28A	0.3170	−0.0398	0.5098	0.071*	
C29A	0.2949 (6)	0.0581 (5)	0.5839 (3)	0.0500 (15)	
H29A	0.2038	0.0868	0.5637	0.060*	
O1B	0.9234 (4)	0.5025 (3)	0.7158 (2)	0.0623 (12)	
N1B	0.7631 (5)	0.6965 (3)	0.7506 (2)	0.0427 (11)	
N2B	0.7236 (4)	0.4144 (3)	0.7293 (2)	0.0413 (11)	
H2B	0.6326	0.4217	0.7315	0.050*	
C1B	0.8992 (6)	0.7412 (4)	0.7392 (3)	0.0485 (15)	
H1B1	0.9780	0.6891	0.7537	0.058*	
H1B2	0.9229	0.8077	0.7647	0.058*	
C2B	0.8889 (5)	0.7660 (4)	0.6694 (3)	0.0410 (13)	
C3B	0.9876 (6)	0.8381 (4)	0.6513 (3)	0.0535 (16)	
H3B	1.0591	0.8712	0.6835	0.064*	
C4B	0.9815 (7)	0.8613 (5)	0.5870 (3)	0.0597 (17)	
H4B	1.0485	0.9103	0.5754	0.072*	
C5B	0.8789 (7)	0.8137 (6)	0.5401 (3)	0.0625 (18)	
H5B	0.8746	0.8290	0.4960	0.075*	
C6B	0.7813 (6)	0.7425 (5)	0.5579 (3)	0.0540 (16)	
H6B	0.7099	0.7095	0.5256	0.065*	
C7B	0.7866 (5)	0.7187 (4)	0.6224 (3)	0.0411 (14)	
C8B	0.6736 (6)	0.6440 (5)	0.6385 (3)	0.0497 (15)	
H8B1	0.6664	0.5799	0.6106	0.060*	
H8B2	0.5790	0.6809	0.6286	0.060*	
C9B	0.7039 (5)	0.6072 (4)	0.7090 (3)	0.0438 (14)	
H9B	0.6086	0.5892	0.7192	0.053*	
C10B	0.7946 (5)	0.5040 (4)	0.7188 (3)	0.0444 (14)	
C11B	0.7836 (5)	0.3064 (4)	0.7372 (3)	0.0388 (14)	
C12B	0.7980 (5)	0.2485 (4)	0.6820 (3)	0.0380 (13)	
C13B	0.8478 (5)	0.1430 (4)	0.6905 (3)	0.0440 (14)	
H13B	0.8625	0.1027	0.6545	0.053*	
C14B	0.8759 (5)	0.0963 (4)	0.7511 (3)	0.0468 (15)	
H14B	0.9048	0.0232	0.7558	0.056*	
C15B	0.8624 (5)	0.1554 (4)	0.8049 (3)	0.0458 (14)	
H15B	0.8857	0.1235	0.8463	0.055*	
C16B	0.8145 (5)	0.2617 (4)	0.7980 (3)	0.0424 (13)	
C17B	0.7624 (5)	0.2969 (4)	0.6148 (3)	0.0448 (14)	
H17B	0.7483	0.3759	0.6193	0.054*	
C18B	0.6219 (7)	0.2505 (7)	0.5757 (3)	0.097 (3)	
H18D	0.6012	0.2824	0.5327	0.145*	
H18E	0.5429	0.2668	0.5973	0.145*	
H18F	0.6314	0.1725	0.5722	0.145*	
C19B	0.8836 (7)	0.2812 (7)	0.5783 (3)	0.091 (3)	
H19D	0.8555	0.3134	0.5355	0.136*	
H19E	0.9012	0.2043	0.5741	0.136*	
H19F	0.9716	0.3158	0.6021	0.136*	
C20B	0.8034 (6)	0.3296 (5)	0.8566 (3)	0.0508 (15)	
H20B	0.7239	0.3827	0.8428	0.061*	
C21B	0.9450 (7)	0.3931 (6)	0.8793 (3)	0.082 (2)	
H21D	0.9377	0.4371	0.9166	0.123*	
H21E	0.9615	0.4396	0.8444	0.123*	
H21F	1.0257	0.3428	0.8912	0.123*	
C22B	0.7676 (8)	0.2633 (6)	0.9117 (3)	0.076 (2)	
H22D	0.7612	0.3109	0.9477	0.115*	
H22E	0.8435	0.2100	0.9259	0.115*	
H22F	0.6751	0.2267	0.8969	0.115*	
C23B	0.7704 (7)	0.6783 (5)	0.8191 (3)	0.0601 (17)	
H23C	0.8641	0.6446	0.8383	0.072*	
H23D	0.6933	0.6274	0.8241	0.072*	
C24B	0.7541 (6)	0.7802 (5)	0.8557 (3)	0.0501 (15)	
C25B	0.8105 (9)	0.7866 (6)	0.9212 (4)	0.084 (2)	
H25B	0.8687	0.7302	0.9428	0.101*	
C26B	0.7811 (11)	0.8770 (8)	0.9553 (4)	0.112 (3)	
H26B	0.8192	0.8812	1.0003	0.135*	
C27B	0.6978 (10)	0.9599 (7)	0.9248 (4)	0.097 (3)	
H27B	0.6766	1.0195	0.9490	0.116*	
C28B	0.6461 (7)	0.9569 (5)	0.8606 (4)	0.0690 (19)	
H28B	0.5914	1.0154	0.8396	0.083*	
C29B	0.6732 (6)	0.8677 (5)	0.8251 (3)	0.0543 (16)	
H29B	0.6369	0.8660	0.7799	0.065*	

### Atomic displacement parameters (Å^2^)

**Table:**

	*U*^11^	*U*^22^	*U*^33^	*U*^12^	*U*^13^	*U*^23^
O1A	0.029 (2)	0.047 (2)	0.097 (3)	0.0012 (17)	0.031 (2)	0.017 (2)
N1A	0.047 (3)	0.032 (2)	0.044 (3)	0.005 (2)	0.017 (2)	0.000 (2)
N2A	0.026 (2)	0.031 (2)	0.065 (3)	0.0003 (19)	0.018 (2)	0.006 (2)
C1A	0.040 (3)	0.039 (3)	0.060 (4)	0.000 (2)	0.013 (3)	0.001 (3)
C2A	0.039 (3)	0.044 (3)	0.045 (4)	−0.002 (3)	0.009 (3)	0.003 (3)
C3A	0.065 (4)	0.044 (3)	0.053 (4)	0.001 (3)	0.021 (3)	−0.002 (3)
C4A	0.106 (6)	0.057 (4)	0.077 (5)	0.001 (4)	0.043 (5)	0.016 (4)
C5A	0.143 (7)	0.061 (5)	0.051 (4)	−0.014 (4)	0.043 (4)	0.007 (4)
C6A	0.095 (5)	0.056 (4)	0.048 (4)	−0.006 (4)	0.023 (4)	−0.002 (3)
C7A	0.053 (4)	0.051 (4)	0.048 (4)	0.001 (3)	0.020 (3)	0.005 (3)
C8A	0.056 (4)	0.045 (3)	0.057 (4)	−0.003 (3)	0.025 (3)	−0.004 (3)
C9A	0.031 (3)	0.040 (3)	0.061 (4)	0.001 (3)	0.021 (3)	0.003 (3)
C10A	0.040 (3)	0.039 (3)	0.054 (4)	−0.002 (3)	0.023 (3)	−0.002 (3)
C11A	0.028 (3)	0.032 (3)	0.056 (4)	0.001 (2)	0.017 (3)	0.007 (3)
C12A	0.038 (3)	0.040 (3)	0.050 (4)	0.002 (3)	0.019 (3)	0.004 (3)
C13A	0.043 (3)	0.046 (3)	0.060 (4)	−0.001 (3)	0.013 (3)	−0.008 (3)
C14A	0.040 (3)	0.037 (3)	0.064 (4)	−0.005 (3)	0.021 (3)	−0.002 (3)
C15A	0.040 (3)	0.043 (3)	0.051 (4)	0.005 (3)	0.018 (3)	0.005 (3)
C16A	0.031 (3)	0.031 (3)	0.058 (4)	0.005 (2)	0.015 (3)	−0.001 (3)
C17A	0.057 (4)	0.054 (4)	0.053 (4)	−0.012 (3)	0.020 (3)	−0.003 (3)
C18A	0.097 (5)	0.098 (6)	0.071 (5)	0.028 (5)	0.040 (4)	0.008 (4)
C19A	0.074 (5)	0.102 (6)	0.072 (5)	0.014 (4)	0.024 (4)	0.017 (4)
C20A	0.034 (3)	0.045 (3)	0.059 (4)	−0.001 (3)	0.018 (3)	−0.005 (3)
C21A	0.064 (4)	0.080 (5)	0.077 (5)	0.014 (4)	−0.010 (4)	−0.023 (4)
C22A	0.061 (4)	0.136 (7)	0.116 (6)	−0.030 (5)	0.048 (4)	−0.076 (6)
C23A	0.040 (3)	0.043 (3)	0.049 (4)	0.003 (3)	0.014 (3)	0.009 (3)
C24A	0.039 (3)	0.033 (3)	0.056 (4)	−0.009 (2)	0.025 (3)	−0.006 (3)
C25A	0.036 (3)	0.046 (3)	0.046 (3)	−0.001 (3)	0.012 (3)	0.003 (3)
C26A	0.046 (3)	0.044 (3)	0.060 (4)	0.008 (3)	0.021 (3)	0.004 (3)
C27A	0.062 (4)	0.053 (4)	0.061 (4)	0.008 (3)	0.024 (4)	−0.001 (3)
C28A	0.071 (4)	0.058 (4)	0.049 (4)	−0.005 (3)	0.014 (3)	−0.012 (3)
C29A	0.043 (3)	0.053 (4)	0.053 (4)	−0.003 (3)	0.010 (3)	0.000 (3)
O1B	0.035 (2)	0.043 (2)	0.115 (4)	0.0009 (19)	0.032 (2)	0.008 (2)
N1B	0.045 (3)	0.046 (3)	0.041 (3)	−0.009 (2)	0.019 (2)	−0.003 (2)
N2B	0.031 (2)	0.032 (2)	0.067 (3)	−0.002 (2)	0.024 (2)	0.000 (2)
C1B	0.039 (3)	0.043 (3)	0.065 (4)	0.001 (3)	0.015 (3)	−0.002 (3)
C2B	0.035 (3)	0.041 (3)	0.048 (4)	0.004 (3)	0.012 (3)	0.000 (3)
C3B	0.040 (3)	0.053 (4)	0.073 (5)	0.006 (3)	0.025 (3)	−0.003 (3)
C4B	0.054 (4)	0.056 (4)	0.078 (5)	0.007 (3)	0.035 (4)	0.017 (4)
C5B	0.060 (4)	0.079 (4)	0.055 (4)	0.017 (4)	0.026 (4)	0.021 (4)
C6B	0.045 (3)	0.064 (4)	0.053 (4)	0.015 (3)	0.009 (3)	−0.007 (3)
C7B	0.035 (3)	0.043 (3)	0.049 (4)	0.009 (2)	0.018 (3)	0.000 (3)
C8B	0.037 (3)	0.046 (3)	0.068 (4)	0.001 (3)	0.015 (3)	−0.007 (3)
C9B	0.028 (3)	0.043 (3)	0.067 (4)	−0.003 (2)	0.025 (3)	−0.006 (3)
C10B	0.030 (3)	0.043 (3)	0.065 (4)	0.000 (3)	0.020 (3)	−0.003 (3)
C11B	0.025 (3)	0.034 (3)	0.061 (4)	0.000 (2)	0.016 (3)	0.000 (3)
C12B	0.029 (3)	0.038 (3)	0.050 (4)	0.000 (2)	0.017 (3)	0.001 (3)
C13B	0.041 (3)	0.037 (3)	0.060 (4)	−0.002 (3)	0.024 (3)	−0.009 (3)
C14B	0.035 (3)	0.040 (3)	0.067 (4)	−0.003 (3)	0.015 (3)	0.003 (3)
C15B	0.038 (3)	0.050 (4)	0.050 (4)	0.000 (3)	0.010 (3)	0.009 (3)
C16B	0.033 (3)	0.044 (3)	0.055 (4)	−0.001 (3)	0.017 (3)	−0.002 (3)
C17B	0.042 (3)	0.039 (3)	0.058 (4)	−0.001 (3)	0.022 (3)	0.002 (3)
C18B	0.067 (5)	0.121 (6)	0.088 (5)	−0.025 (5)	−0.014 (4)	0.055 (5)
C19B	0.069 (5)	0.146 (7)	0.070 (5)	0.027 (5)	0.043 (4)	0.031 (5)
C20B	0.047 (4)	0.054 (4)	0.056 (4)	0.001 (3)	0.020 (3)	−0.004 (3)
C21B	0.070 (5)	0.099 (5)	0.077 (5)	−0.020 (4)	0.019 (4)	−0.034 (5)
C22B	0.104 (6)	0.079 (5)	0.054 (4)	0.010 (4)	0.035 (4)	0.009 (4)
C23B	0.062 (4)	0.057 (4)	0.067 (4)	0.007 (3)	0.027 (3)	0.014 (4)
C24B	0.054 (4)	0.057 (4)	0.045 (4)	−0.015 (3)	0.025 (3)	−0.001 (3)
C25B	0.108 (6)	0.076 (5)	0.066 (5)	−0.010 (4)	0.014 (5)	0.015 (5)
C26B	0.208 (11)	0.090 (6)	0.035 (4)	−0.020 (7)	0.016 (6)	0.003 (5)
C27B	0.174 (9)	0.069 (5)	0.058 (5)	−0.023 (6)	0.047 (5)	−0.002 (4)
C28B	0.080 (5)	0.064 (4)	0.069 (5)	−0.014 (4)	0.030 (4)	−0.014 (4)
C29B	0.057 (4)	0.055 (4)	0.053 (4)	−0.004 (3)	0.016 (3)	−0.010 (3)

### Geometric parameters (Å, °)

**Table:**

O1A—C10A	1.238 (6)	O1B—C10B	1.239 (5)
N1A—C1A	1.456 (6)	N1B—C9B	1.458 (7)
N1A—C23A	1.470 (6)	N1B—C23B	1.463 (7)
N1A—C9A	1.475 (6)	N1B—C1B	1.474 (6)
N2A—C10A	1.341 (6)	N2B—C10B	1.347 (6)
N2A—C11A	1.435 (6)	N2B—C11B	1.457 (6)
N2A—H2A	0.8800	N2B—H2B	0.8800
C1A—C2A	1.502 (7)	C1B—C2B	1.499 (7)
C1A—H1A1	0.9900	C1B—H1B1	0.9900
C1A—H1A2	0.9900	C1B—H1B2	0.9900
C2A—C7A	1.379 (8)	C2B—C7B	1.371 (7)
C2A—C3A	1.397 (7)	C2B—C3B	1.410 (7)
C3A—C4A	1.387 (8)	C3B—C4B	1.388 (8)
C3A—H3A	0.9500	C3B—H3B	0.9500
C4A—C5A	1.394 (9)	C4B—C5B	1.373 (9)
C4A—H4A	0.9500	C4B—H4B	0.9500
C5A—C6A	1.390 (9)	C5B—C6B	1.393 (8)
C5A—H5A	0.9500	C5B—H5B	0.9500
C6A—C7A	1.392 (8)	C6B—C7B	1.393 (8)
C6A—H6A	0.9500	C6B—H6B	0.9500
C7A—C8A	1.508 (8)	C7B—C8B	1.514 (7)
C8A—C9A	1.535 (7)	C8B—C9B	1.536 (8)
C8A—H8A1	0.9900	C8B—H8B1	0.9900
C8A—H8A2	0.9900	C8B—H8B2	0.9900
C9A—C10A	1.516 (7)	C9B—C10B	1.537 (7)
C9A—H9A	1.0000	C9B—H9B	1.0000
C11A—C16A	1.392 (7)	C11B—C16B	1.382 (7)
C11A—C12A	1.395 (8)	C11B—C12B	1.410 (7)
C12A—C13A	1.397 (7)	C12B—C13B	1.397 (7)
C12A—C17A	1.547 (7)	C12B—C17B	1.523 (7)
C13A—C14A	1.385 (8)	C13B—C14B	1.387 (7)
C13A—H13A	0.9500	C13B—H13B	0.9500
C14A—C15A	1.381 (7)	C14B—C15B	1.389 (7)
C14A—H14A	0.9500	C14B—H14B	0.9500
C15A—C16A	1.408 (7)	C15B—C16B	1.398 (8)
C15A—H15A	0.9500	C15B—H15B	0.9500
C16A—C20A	1.506 (7)	C16B—C20B	1.528 (7)
C17A—C19A	1.518 (9)	C17B—C18B	1.528 (8)
C17A—C18A	1.533 (8)	C17B—C19B	1.530 (7)
C17A—H17A	1.0000	C17B—H17B	1.0000
C18A—H18A	0.9800	C18B—H18D	0.9800
C18A—H18B	0.9800	C18B—H18E	0.9800
C18A—H18C	0.9800	C18B—H18F	0.9800
C19A—H19A	0.9800	C19B—H19D	0.9800
C19A—H19B	0.9800	C19B—H19E	0.9800
C19A—H19C	0.9800	C19B—H19F	0.9800
C20A—C22A	1.510 (7)	C20B—C22B	1.532 (8)
C20A—C21A	1.518 (8)	C20B—C21B	1.546 (8)
C20A—H20A	1.0000	C20B—H20B	1.0000
C21A—H21A	0.9800	C21B—H21D	0.9800
C21A—H21B	0.9800	C21B—H21E	0.9800
C21A—H21C	0.9800	C21B—H21F	0.9800
C22A—H22A	0.9800	C22B—H22D	0.9800
C22A—H22B	0.9800	C22B—H22E	0.9800
C22A—H22C	0.9800	C22B—H22F	0.9800
C23A—C24A	1.510 (7)	C23B—C24B	1.514 (8)
C23A—H23A	0.9900	C23B—H23C	0.9900
C23A—H23B	0.9900	C23B—H23D	0.9900
C24A—C25A	1.403 (7)	C24B—C25B	1.386 (9)
C24A—C29A	1.410 (8)	C24B—C29B	1.411 (8)
C25A—C26A	1.383 (7)	C25B—C26B	1.399 (11)
C25A—H25A	0.9500	C25B—H25B	0.9500
C26A—C27A	1.360 (8)	C26B—C27B	1.377 (12)
C26A—H26A	0.9500	C26B—H26B	0.9500
C27A—C28A	1.358 (8)	C27B—C28B	1.351 (9)
C27A—H27A	0.9500	C27B—H27B	0.9500
C28A—C29A	1.396 (8)	C28B—C29B	1.398 (8)
C28A—H28A	0.9500	C28B—H28B	0.9500
C29A—H29A	0.9500	C29B—H29B	0.9500
			
C1A—N1A—C23A	110.5 (4)	C9B—N1B—C23B	114.7 (4)
C1A—N1A—C9A	110.3 (4)	C9B—N1B—C1B	115.2 (4)
C23A—N1A—C9A	110.8 (4)	C23B—N1B—C1B	111.4 (4)
C10A—N2A—C11A	122.6 (4)	C10B—N2B—C11B	126.0 (4)
C10A—N2A—H2A	118.7	C10B—N2B—H2B	117.0
C11A—N2A—H2A	118.7	C11B—N2B—H2B	117.0
N1A—C1A—C2A	115.3 (4)	N1B—C1B—C2B	111.5 (4)
N1A—C1A—H1A1	108.4	N1B—C1B—H1B1	109.3
C2A—C1A—H1A1	108.4	C2B—C1B—H1B1	109.3
N1A—C1A—H1A2	108.4	N1B—C1B—H1B2	109.3
C2A—C1A—H1A2	108.4	C2B—C1B—H1B2	109.3
H1A1—C1A—H1A2	107.5	H1B1—C1B—H1B2	108.0
C7A—C2A—C3A	118.9 (5)	C7B—C2B—C3B	118.9 (5)
C7A—C2A—C1A	121.2 (5)	C7B—C2B—C1B	121.5 (5)
C3A—C2A—C1A	119.9 (5)	C3B—C2B—C1B	119.7 (5)
C4A—C3A—C2A	121.4 (6)	C4B—C3B—C2B	120.8 (6)
C4A—C3A—H3A	119.3	C4B—C3B—H3B	119.6
C2A—C3A—H3A	119.3	C2B—C3B—H3B	119.6
C3A—C4A—C5A	119.0 (6)	C5B—C4B—C3B	120.1 (6)
C3A—C4A—H4A	120.5	C5B—C4B—H4B	120.0
C5A—C4A—H4A	120.5	C3B—C4B—H4B	120.0
C6A—C5A—C4A	120.0 (6)	C4B—C5B—C6B	119.2 (6)
C6A—C5A—H5A	120.0	C4B—C5B—H5B	120.4
C4A—C5A—H5A	120.0	C6B—C5B—H5B	120.4
C5A—C6A—C7A	120.2 (6)	C5B—C6B—C7B	121.1 (6)
C5A—C6A—H6A	119.9	C5B—C6B—H6B	119.4
C7A—C6A—H6A	119.9	C7B—C6B—H6B	119.4
C2A—C7A—C6A	120.5 (5)	C2B—C7B—C6B	120.0 (5)
C2A—C7A—C8A	119.7 (5)	C2B—C7B—C8B	121.6 (5)
C6A—C7A—C8A	119.8 (5)	C6B—C7B—C8B	118.4 (5)
C7A—C8A—C9A	111.7 (5)	C7B—C8B—C9B	114.2 (4)
C7A—C8A—H8A1	109.3	C7B—C8B—H8B1	108.7
C9A—C8A—H8A1	109.3	C9B—C8B—H8B1	108.7
C7A—C8A—H8A2	109.3	C7B—C8B—H8B2	108.7
C9A—C8A—H8A2	109.3	C9B—C8B—H8B2	108.7
H8A1—C8A—H8A2	107.9	H8B1—C8B—H8B2	107.6
N1A—C9A—C10A	113.0 (4)	N1B—C9B—C8B	109.5 (4)
N1A—C9A—C8A	108.2 (4)	N1B—C9B—C10B	115.1 (4)
C10A—C9A—C8A	107.9 (4)	C8B—C9B—C10B	111.5 (5)
N1A—C9A—H9A	109.2	N1B—C9B—H9B	106.8
C10A—C9A—H9A	109.2	C8B—C9B—H9B	106.8
C8A—C9A—H9A	109.2	C10B—C9B—H9B	106.8
O1A—C10A—N2A	121.8 (5)	O1B—C10B—N2B	122.0 (5)
O1A—C10A—C9A	121.4 (5)	O1B—C10B—C9B	122.4 (5)
N2A—C10A—C9A	116.7 (4)	N2B—C10B—C9B	115.5 (4)
C16A—C11A—C12A	123.2 (5)	C16B—C11B—C12B	122.4 (5)
C16A—C11A—N2A	118.7 (5)	C16B—C11B—N2B	118.8 (5)
C12A—C11A—N2A	118.1 (5)	C12B—C11B—N2B	118.7 (5)
C11A—C12A—C13A	117.7 (5)	C13B—C12B—C11B	117.6 (5)
C11A—C12A—C17A	122.6 (5)	C13B—C12B—C17B	119.8 (5)
C13A—C12A—C17A	119.7 (5)	C11B—C12B—C17B	122.7 (4)
C14A—C13A—C12A	120.7 (6)	C14B—C13B—C12B	120.6 (5)
C14A—C13A—H13A	119.7	C14B—C13B—H13B	119.7
C12A—C13A—H13A	119.7	C12B—C13B—H13B	119.7
C15A—C14A—C13A	120.3 (5)	C13B—C14B—C15B	120.7 (5)
C15A—C14A—H14A	119.8	C13B—C14B—H14B	119.7
C13A—C14A—H14A	119.8	C15B—C14B—H14B	119.7
C14A—C15A—C16A	121.1 (5)	C14B—C15B—C16B	120.1 (5)
C14A—C15A—H15A	119.5	C14B—C15B—H15B	120.0
C16A—C15A—H15A	119.5	C16B—C15B—H15B	120.0
C11A—C16A—C15A	117.0 (5)	C11B—C16B—C15B	118.6 (5)
C11A—C16A—C20A	123.4 (4)	C11B—C16B—C20B	120.2 (5)
C15A—C16A—C20A	119.7 (5)	C15B—C16B—C20B	121.1 (5)
C19A—C17A—C18A	111.4 (5)	C12B—C17B—C18B	110.8 (4)
C19A—C17A—C12A	112.2 (5)	C12B—C17B—C19B	112.6 (5)
C18A—C17A—C12A	113.0 (5)	C18B—C17B—C19B	109.9 (6)
C19A—C17A—H17A	106.6	C12B—C17B—H17B	107.8
C18A—C17A—H17A	106.6	C18B—C17B—H17B	107.8
C12A—C17A—H17A	106.6	C19B—C17B—H17B	107.8
C17A—C18A—H18A	109.5	C17B—C18B—H18D	109.5
C17A—C18A—H18B	109.5	C17B—C18B—H18E	109.5
H18A—C18A—H18B	109.5	H18D—C18B—H18E	109.5
C17A—C18A—H18C	109.5	C17B—C18B—H18F	109.5
H18A—C18A—H18C	109.5	H18D—C18B—H18F	109.5
H18B—C18A—H18C	109.5	H18E—C18B—H18F	109.5
C17A—C19A—H19A	109.5	C17B—C19B—H19D	109.5
C17A—C19A—H19B	109.5	C17B—C19B—H19E	109.5
H19A—C19A—H19B	109.5	H19D—C19B—H19E	109.5
C17A—C19A—H19C	109.5	C17B—C19B—H19F	109.5
H19A—C19A—H19C	109.5	H19D—C19B—H19F	109.5
H19B—C19A—H19C	109.5	H19E—C19B—H19F	109.5
C16A—C20A—C22A	112.1 (4)	C16B—C20B—C22B	113.1 (5)
C16A—C20A—C21A	111.2 (4)	C16B—C20B—C21B	109.4 (4)
C22A—C20A—C21A	111.5 (6)	C22B—C20B—C21B	110.8 (5)
C16A—C20A—H20A	107.2	C16B—C20B—H20B	107.8
C22A—C20A—H20A	107.2	C22B—C20B—H20B	107.8
C21A—C20A—H20A	107.2	C21B—C20B—H20B	107.8
C20A—C21A—H21A	109.5	C20B—C21B—H21D	109.5
C20A—C21A—H21B	109.5	C20B—C21B—H21E	109.5
H21A—C21A—H21B	109.5	H21D—C21B—H21E	109.5
C20A—C21A—H21C	109.5	C20B—C21B—H21F	109.5
H21A—C21A—H21C	109.5	H21D—C21B—H21F	109.5
H21B—C21A—H21C	109.5	H21E—C21B—H21F	109.5
C20A—C22A—H22A	109.5	C20B—C22B—H22D	109.5
C20A—C22A—H22B	109.5	C20B—C22B—H22E	109.5
H22A—C22A—H22B	109.5	H22D—C22B—H22E	109.5
C20A—C22A—H22C	109.5	C20B—C22B—H22F	109.5
H22A—C22A—H22C	109.5	H22D—C22B—H22F	109.5
H22B—C22A—H22C	109.5	H22E—C22B—H22F	109.5
N1A—C23A—C24A	112.4 (4)	N1B—C23B—C24B	113.2 (5)
N1A—C23A—H23A	109.1	N1B—C23B—H23C	108.9
C24A—C23A—H23A	109.1	C24B—C23B—H23C	108.9
N1A—C23A—H23B	109.1	N1B—C23B—H23D	108.9
C24A—C23A—H23B	109.1	C24B—C23B—H23D	108.9
H23A—C23A—H23B	107.9	H23C—C23B—H23D	107.7
C25A—C24A—C29A	118.2 (5)	C25B—C24B—C29B	118.5 (6)
C25A—C24A—C23A	122.4 (5)	C25B—C24B—C23B	120.4 (6)
C29A—C24A—C23A	119.3 (5)	C29B—C24B—C23B	121.0 (5)
C26A—C25A—C24A	119.5 (5)	C24B—C25B—C26B	119.4 (7)
C26A—C25A—H25A	120.2	C24B—C25B—H25B	120.3
C24A—C25A—H25A	120.2	C26B—C25B—H25B	120.3
C27A—C26A—C25A	121.8 (5)	C27B—C26B—C25B	121.1 (7)
C27A—C26A—H26A	119.1	C27B—C26B—H26B	119.4
C25A—C26A—H26A	119.1	C25B—C26B—H26B	119.4
C28A—C27A—C26A	119.8 (6)	C28B—C27B—C26B	120.4 (7)
C28A—C27A—H27A	120.1	C28B—C27B—H27B	119.8
C26A—C27A—H27A	120.1	C26B—C27B—H27B	119.8
C27A—C28A—C29A	120.9 (6)	C27B—C28B—C29B	119.9 (7)
C27A—C28A—H28A	119.6	C27B—C28B—H28B	120.0
C29A—C28A—H28A	119.6	C29B—C28B—H28B	120.0
C28A—C29A—C24A	119.7 (5)	C28B—C29B—C24B	120.6 (6)
C28A—C29A—H29A	120.2	C28B—C29B—H29B	119.7
C24A—C29A—H29A	120.2	C24B—C29B—H29B	119.7
			
C23A—N1A—C1A—C2A	−167.1 (4)	C9B—N1B—C1B—C2B	50.4 (6)
C9A—N1A—C1A—C2A	−44.3 (6)	C23B—N1B—C1B—C2B	−176.6 (4)
N1A—C1A—C2A—C7A	10.2 (7)	N1B—C1B—C2B—C7B	−20.6 (7)
N1A—C1A—C2A—C3A	−171.9 (5)	N1B—C1B—C2B—C3B	160.4 (4)
C7A—C2A—C3A—C4A	−0.7 (9)	C7B—C2B—C3B—C4B	0.2 (7)
C1A—C2A—C3A—C4A	−178.7 (5)	C1B—C2B—C3B—C4B	179.2 (5)
C2A—C3A—C4A—C5A	−0.8 (10)	C2B—C3B—C4B—C5B	−0.2 (8)
C3A—C4A—C5A—C6A	1.4 (11)	C3B—C4B—C5B—C6B	0.3 (9)
C4A—C5A—C6A—C7A	−0.5 (11)	C4B—C5B—C6B—C7B	−0.2 (9)
C3A—C2A—C7A—C6A	1.7 (8)	C3B—C2B—C7B—C6B	−0.2 (7)
C1A—C2A—C7A—C6A	179.6 (5)	C1B—C2B—C7B—C6B	−179.1 (5)
C3A—C2A—C7A—C8A	−176.7 (5)	C3B—C2B—C7B—C8B	−177.5 (4)
C1A—C2A—C7A—C8A	1.3 (8)	C1B—C2B—C7B—C8B	3.5 (7)
C5A—C6A—C7A—C2A	−1.1 (10)	C5B—C6B—C7B—C2B	0.2 (8)
C5A—C6A—C7A—C8A	177.2 (6)	C5B—C6B—C7B—C8B	177.6 (5)
C2A—C7A—C8A—C9A	20.9 (7)	C2B—C7B—C8B—C9B	−12.9 (7)
C6A—C7A—C8A—C9A	−157.4 (5)	C6B—C7B—C8B—C9B	169.7 (5)
C1A—N1A—C9A—C10A	−174.4 (5)	C23B—N1B—C9B—C8B	168.6 (4)
C23A—N1A—C9A—C10A	−51.8 (6)	C1B—N1B—C9B—C8B	−60.0 (5)
C1A—N1A—C9A—C8A	66.2 (5)	C23B—N1B—C9B—C10B	−64.9 (6)
C23A—N1A—C9A—C8A	−171.2 (4)	C1B—N1B—C9B—C10B	66.5 (6)
C7A—C8A—C9A—N1A	−53.9 (5)	C7B—C8B—C9B—N1B	39.0 (6)
C7A—C8A—C9A—C10A	−176.5 (4)	C7B—C8B—C9B—C10B	−89.4 (5)
C11A—N2A—C10A—O1A	−6.0 (8)	C11B—N2B—C10B—O1B	−1.1 (9)
C11A—N2A—C10A—C9A	169.5 (5)	C11B—N2B—C10B—C9B	177.3 (5)
N1A—C9A—C10A—O1A	−48.6 (7)	N1B—C9B—C10B—O1B	−54.7 (7)
C8A—C9A—C10A—O1A	71.0 (6)	C8B—C9B—C10B—O1B	70.7 (7)
N1A—C9A—C10A—N2A	135.9 (5)	N1B—C9B—C10B—N2B	126.9 (5)
C8A—C9A—C10A—N2A	−104.5 (5)	C8B—C9B—C10B—N2B	−107.7 (5)
C10A—N2A—C11A—C16A	94.7 (6)	C10B—N2B—C11B—C16B	105.7 (6)
C10A—N2A—C11A—C12A	−83.8 (6)	C10B—N2B—C11B—C12B	−78.9 (6)
C16A—C11A—C12A—C13A	−0.5 (7)	C16B—C11B—C12B—C13B	−1.1 (7)
N2A—C11A—C12A—C13A	177.9 (4)	N2B—C11B—C12B—C13B	−176.3 (4)
C16A—C11A—C12A—C17A	−179.3 (5)	C16B—C11B—C12B—C17B	179.3 (4)
N2A—C11A—C12A—C17A	−0.9 (7)	N2B—C11B—C12B—C17B	4.0 (7)
C11A—C12A—C13A—C14A	1.4 (7)	C11B—C12B—C13B—C14B	2.6 (7)
C17A—C12A—C13A—C14A	−179.7 (5)	C17B—C12B—C13B—C14B	−177.7 (4)
C12A—C13A—C14A—C15A	−0.4 (8)	C12B—C13B—C14B—C15B	−3.4 (8)
C13A—C14A—C15A—C16A	−1.7 (8)	C13B—C14B—C15B—C16B	2.5 (8)
C12A—C11A—C16A—C15A	−1.5 (7)	C12B—C11B—C16B—C15B	0.3 (7)
N2A—C11A—C16A—C15A	−179.9 (4)	N2B—C11B—C16B—C15B	175.5 (4)
C12A—C11A—C16A—C20A	−179.8 (4)	C12B—C11B—C16B—C20B	177.3 (4)
N2A—C11A—C16A—C20A	1.8 (7)	N2B—C11B—C16B—C20B	−7.5 (7)
C14A—C15A—C16A—C11A	2.5 (7)	C14B—C15B—C16B—C11B	−1.0 (7)
C14A—C15A—C16A—C20A	−179.0 (4)	C14B—C15B—C16B—C20B	−177.9 (5)
C11A—C12A—C17A—C19A	101.6 (6)	C13B—C12B—C17B—C18B	73.6 (6)
C13A—C12A—C17A—C19A	−77.2 (7)	C11B—C12B—C17B—C18B	−106.8 (6)
C11A—C12A—C17A—C18A	−131.5 (6)	C13B—C12B—C17B—C19B	−49.9 (7)
C13A—C12A—C17A—C18A	49.6 (7)	C11B—C12B—C17B—C19B	129.7 (6)
C11A—C16A—C20A—C22A	−122.6 (6)	C11B—C16B—C20B—C22B	151.7 (5)
C15A—C16A—C20A—C22A	59.1 (7)	C15B—C16B—C20B—C22B	−31.3 (7)
C11A—C16A—C20A—C21A	111.8 (6)	C11B—C16B—C20B—C21B	−84.2 (6)
C15A—C16A—C20A—C21A	−66.5 (6)	C15B—C16B—C20B—C21B	92.7 (6)
C1A—N1A—C23A—C24A	−64.0 (5)	C9B—N1B—C23B—C24B	−149.5 (5)
C9A—N1A—C23A—C24A	173.6 (4)	C1B—N1B—C23B—C24B	77.3 (6)
N1A—C23A—C24A—C25A	−37.8 (6)	N1B—C23B—C24B—C25B	−155.5 (6)
N1A—C23A—C24A—C29A	146.7 (5)	N1B—C23B—C24B—C29B	28.9 (8)
C29A—C24A—C25A—C26A	−1.1 (7)	C29B—C24B—C25B—C26B	2.5 (10)
C23A—C24A—C25A—C26A	−176.6 (4)	C23B—C24B—C25B—C26B	−173.2 (7)
C24A—C25A—C26A—C27A	1.5 (7)	C24B—C25B—C26B—C27B	−0.4 (13)
C25A—C26A—C27A—C28A	−0.7 (8)	C25B—C26B—C27B—C28B	−1.9 (14)
C26A—C27A—C28A—C29A	−0.6 (9)	C26B—C27B—C28B—C29B	2.1 (12)
C27A—C28A—C29A—C24A	1.0 (8)	C27B—C28B—C29B—C24B	0.1 (9)
C25A—C24A—C29A—C28A	−0.2 (7)	C25B—C24B—C29B—C28B	−2.5 (9)
C23A—C24A—C29A—C28A	175.5 (5)	C23B—C24B—C29B—C28B	173.2 (5)

### Hydrogen-bond geometry (Å, °)

**Table:**

*D*—H···*A*	*D*—H	H···*A*	*D*···*A*	*D*—H···*A*
N2A—H2A···O1B^i^	0.88	2.15	2.900 (6)	142

Symmetry codes: (i) *x*−1, *y*, *z*.

## References

[bb1] Bruker (2006). *APEX2*, *SADABS* and *SAINT* Bruker AXS Inc., Madison, Wisconsin, USA.

[bb2] Dolomanov, O. V., Bourhis, L. J., Gildea, R. J., Howard, J. A. K. & Puschmann, H. (2009). *J. Appl. Cryst.* **42**, 339–341.

[bb3] Naicker, T., Govender, T., Kruger, H. G. & Maguire, G. E. M. (2011). *Acta Cryst.* E**67**, o67. 10.1107/S1600536810050361 PMC3050362 21522778

[bb4] Naicker, T., Petzold, K., Singh, T., Arvidsson, P. I., Kruger, H. G., Maguire, G. E. M. & Govender, T. (2010). *Tetrahedron Asymmetry*, **21**, 2859–2867.

[bb5] Sheldrick, G. M. (2008). *Acta Cryst.* A**64**, 112–122. 10.1107/S0108767307043930 18156677

